# ^18^F-FDG PET/CT imaging findings in anaplastic large cell lymphoma, a rare subtype of lymphoma

**DOI:** 10.1186/s40644-019-0278-5

**Published:** 2020-01-10

**Authors:** Yanping Jiang, Lijuan Wang, Wenlan Zhou, Jiamei Gu, Ying Tian, Ye Dong, Lilan Fu, Hu-bing Wu

**Affiliations:** Nanfang PET Center, Nanfang Hospital, Southern Medical University, 1838 Guangzhou Avenue North, Guangzhou, 510515 Guangdong Province China

**Keywords:** Anaplastic large cell lymphoma (ALCL), Anaplastic lymphoma kinase (ALK), Diffuse large B cell lymphoma (DLBCL), Positron emission tomography (PET), Computed tomography (CT), Deoxyglucose, Image feature

## Abstract

**Objective:**

To investigate the ^18^F-FDG PET/CT imaging manifestations for anaplastic large cell lymphoma (ALCL), a rare subtype of T/NK cell lymphoma.

**Methods:**

Fifty patients with ALCL, including 32 anaplastic lymphoma kinase (ALK)-positive patients and 18 ALK-negative patients, were enrolled. The positive detection, maximal standardized uptake value (SUV_max_), and distribution of nodal and extranodal involvement were recorded and analysed. Fifty patients with diffuse large B cell lymphoma (DLBCL) were collected as a control group.

**Results:**

ALCL lesions were demonstrated to be ^18^F-FDG-avid tumours with a mean SUVmax of 19.4 ± 12.6. Most (76%) ALCL patients presented with stage III-IV disease, and nodal and extranodal involvement occurred in 74.0 and 72.0% of the patients, respectively. ALCL and DLBCL showed many similarities in tumour stage, ^18^F-FDG uptake and tumour involvement (*P* > 0.05), although the preferred extranodal organs of involvement (bone and the gastrointestinal tract, respectively) were different (*P* < 0.05). Compared to ALK-negative lesions, a higher uptake of ^18^F-FDG was found in the ALK-positive lesions (SUVmax: 22.1 ± 14.3 vs. 15.1 ± 6.6, *t* = 2.354, *P* = 0.023). ALK-positive ALCL was more likely to involve the lymph nodes than ALK-negative ALCL (84.3% vs. 55.5%, χ^2^ = 4.973, *P* = 0.043), while ALK-negative ALCL was more prone to involve the extranodal organs compared to ALK-positive ALCL (88.9% vs. 62.5%, χ^2^ = 3.979, *P* = 0.046).

**Conclusion:**

The present study demonstrated that ALCL is a systemic ^18^F-FDG-avid lymphoma with many imaging manifestations similar to DLBCL on PET/CT. The present study also showed that ALK expression actually influenced tumour ^18^F-FDG uptake and lesion distribution. These findings may be useful to improve the understanding of the biological characteristics of ALCL.

## Introduction

Anaplastic large cell lymphoma (ALCL) is a rare CD30^+^ T-cell subtype of non-Hodgkin’s lymphoma (NHL). The frequency of this disease varies geographically, with the highest incidence in parts of Asia, including China; however, it is particularly rare in European and America [1–3]. Since Stein et al. first reported it in 1985, ALCL has been studied for clinical features, treatment and prognosis over the years [4]. Two subtypes of systemic ALCL are recognized by the World Health Organization (WHO) classification system according to the expression of anaplastic lymphoma kinase (ALK): ALK-positive ALCL and ALK-negative ALCL. ALK-positive ALCL is associated with translocations involving *ALK*, the **A**naplastic **L**ymphoma **K**inase gene, located on chromosome 2p23. As an independent prognostic index, ALCL with positive expression of ALK is more responsive to therapy and associated with a better prognosis than ALK-negative ALCL [1, 3, 5–7].

^18^F-FDG positron emission tomography/computed tomography (PET/CT) is an important functional imaging technique that has become a standard imaging modality in staging, treatment response evaluation and prognostic prediction for FDG-avid lymphomas, according to the updated guidelines for imaging in lymphoma [8]. However, the imaging characteristics of ALCL on ^18^F-FDG PET/CT have rarely been described, and the role of ^18^F-FDG PET/CT in evaluating this disease has not been well established, possibly due to the low incidence rate of the disease [9]. In the present study, ^18^F-FDG PET/CT images of 50 patients with ALCL were retrospectively reviewed to describe the ALCL imaging manifestation. Fifty patients with DLBCL, the most common subtype of lymphoma, were selected as the control group to compare the differences between the two diseases.

## Materials and methods

### Patients

The institutional review board of Nanfang Hospital, Southern Medical University, approved this retrospective study. The institutional review board waived the requirement for written informed consent because of the retrospective nature of the study.

Fifty consecutive patients with histopathologically confirmed ALCL who received ^18^F-FDG PET/CT for staging from November 2005 to April 2018 were enrolled in the present study. If an ALCL patient received anti-tumour therapy, had uncontrolled diabetes, or had a history of other malignancies, he or she was excluded from the present study. Clinical data were collected for all of the patients, including age, sex, Ann Arbor clinical stage and the presence of B symptoms (fevers, night sweats and weight loss). ^18^F-FDG PET/CT images were retrospectively reviewed with a special focus on tumour ^18^F-FDG uptake and the distribution of nodal and extranodal involvement. To compare the difference in imaging manifestations of ALCL on ^18^F-FDG PET/CT to DLBCL, 50 consecutive patients with DLBCL who received ^18^F-FDG PET/CT for staging from July 2017 to September 2018 were collected as a control group.

1. **2** ^**18**^**F-FDG PET/CT**

^18^F-FDG PET/CT scan was performed using a Biograph mCTx scanner (Siemens, Germany) in 47 patients and using a Discovery LS (GE, America) in 3 additional patients. The patients were instructed to fast for at least 5 h and had blood glucose levels less than 11.1 mmol/L. Whole-body PET/CT was performed approximately 60 min after the intravenous injection of 222–446 MBq (6.0~12.1 mCi, 150 μCi/kg) ^18^F-FDG. CT data were acquired using the following parameters: voltage (120 kV for Biograph mCTx scanner, 140 kV for Discovery LS scanner), current (automatic milliampere-ampere), pitch (0.55), single-coil rotation time (1.0 s) and layer thickness (3 mm). 3D PET data were then acquired with a 2 min/bed position for the Biograph mCTx scanner, and 2D PET data were then acquired with a 4 min/bed position for the Discovery LS scanner according the established protocol in our centre [10, 11]*.*

PET data were reconstructed using the ordered subset maximum expectation iterative method (OSEM) with CT information for attenuation correction. PET and CT images were transferred to the Xeleris or Syngo MMWP workstations for image alignment fusion and analysis. The PET, CT and fused PET/CT data were independently reviewed by two experienced nuclear medical physicians who were blinded to the expression of ALK status and other clinical data. Any initial difference of opinion was resolved by consensus. Lesions with abnormally increased uptake of ^18^F-FDG, i.e., higher than the radioactivity in the mediastinal blood pool specifically in the aortic arch, were considered positive. A region of interest (ROI) was placed over the lesions to measure each SUVmax. Based on the patient-specific lymphoma involvement, the lesion with the highest uptake of ^18^F-FDG was selected as the target lesion for SUVmax analysis. For the lesion-based/district-based analysis, the sites of lymph node involvement were partitioned into the neck, supraclavicular fossa, axilla, hilar of lung, mediastinum, retroperitoneal area, parailiac common vessels, internal and external parailiac vessels, inguinal region, and upper and lower limbs. Each site was scored as “1”. For those sites with bilateral sides, positive lymph nodes found on one side were scored as “1”, while those on both sides were scored as “2”. Single site, multisite and widespread involvement was used to classify the lymph node involvement into a single site, 2~5 sites and more than 5 sites of positive lymph nodes.

The positive findings on PET and on the CT of PET/CT were comparatively analysed, especially for extranodal involvement. The CT scans were acquired under a non-enhanced contrast condition. On the PET images, a focal uptake of ^18^F-FDG was considered a positive lesion for lymphoma, while on the CT images, a solid nodule, or mass, or thickened tissue were considered positive lesion.

### Immunohistopathology of ALK expression

According to the WHO classification diagnostic standard, the diagnosis of ALCL is established on morphologic analysis and immunohistochemical examination. The tumour is characterized by the presence of large cells with horseshoe-shaped nuclei, prominent nucleoli, with or without a paranuclear HOF growing in a cohesive growth pattern, strong and homogeneous CD30 expression in the membrane and a Golgi pattern on immunohistochemical analysis. The expression of ALK in the ALCLs was measured by FISH analysis using a Vysis ALK gene rearrangement assay kit. FISH results were evaluated according to the number of positive cells. ALK-positive staining was defined as positive when 25 per 50 cells were positively stained. When the specimens had between 5 and 25 positive cells, the number of counted cells increased to 100 cells, and ALK-positivity was defined by more than 50 positive cells. For the Vysis ALK gene rearrangement assay, if there were ≥ 15 positive cells, ALK rearrangement was considered positive. If there were less than 15 positive cells, the result was considered negative.

### Statistical analysis

IBM SPSS 19.0 software was used for the data analysis. Measurement data that conformed to the normal distribution were expressed as x ± s. Independent two-sample *t* tests were used to compare the SUV_max_ between two groups. The quantitative staging difference, nodal involvement, lymph node lesion distribution, and extranodal organ involvement data were expressed in frequencies or percentages. Pearson chi-square tests or Fisher’s exact tests were used for the data analysis. A *P* value less than 0.05 was defined as statistically significant.

## Results

### Clinical characteristics of the patients with ALCL

Of the 50 patients with ALCL, 32 were positive for ALK and 18 were negative for ALK, with a male to female ratio of 2.6:1 and a median age of 28 years (range from 3 to 76 years old). According to the Ann Arbor staging system, 12 patients were classified as stage I-II (24%), and 38 patients were classified as stage III-IV (76%); 34 patients (68%) had B symptoms.

### ^18^F-FDG PET/CT findings in the patients with ALCL

PET/CT detected ^18^F-FDG-avid lymphoma lesions in at least one site in 49 out of 50 patients with a positive detection rate of 98.0%. A false-negative result was observed in a 3-year-old boy who had a large tumour (largest diameter, 7.0 cm) in the abdomen with a mild uptake of ^18^F-FDG (SUVmax, 1.3). The mean SUVmax of the ALCL lesions was 19.4 ± 12.6. Nodal involvement was found in 37 (74.0%) ALCL patients, and extranodal involvement was found in 36 ALCL patients (72.0%). On the PET/CT images, 11 patients presented with solitary tumours, while the other 39 patients had multiple or widespread lesions. Of the 50 ALCL patients, 23 (46%) had both nodal and extranodal involvement, whereas 14 patients (28%) had nodal involvement alone and 13 patients (26%) had extranodal involvement alone. The most common nodal involved zone was the neck, followed by the mediastinum, retroperitoneal zone, mesentery and peri-iliac zone. The most commonly involved extranodal organ was bone (22/50), followed by the skin (8/50), spleen (6/50) and parotid gland (6/50). ^18^F-FDG PET was demonstrated to be superior to CT for detecting extranodal involvement, notably bone involvement. The positive detection rate of PET was 100% for bone involvement, which was significantly higher than the 59.1% positive detection rate of CT (χ^2^ = 11.314, *P* = 0.001). PET also showed more sensitivity in the detection of cutaneous involvement than CT (8/8 vs. 3/8, χ^2^ = 7.273, *P* = 0.026).

### Difference in the imaging manifestations between ALCL and DLBCL

**Fig. 1:**
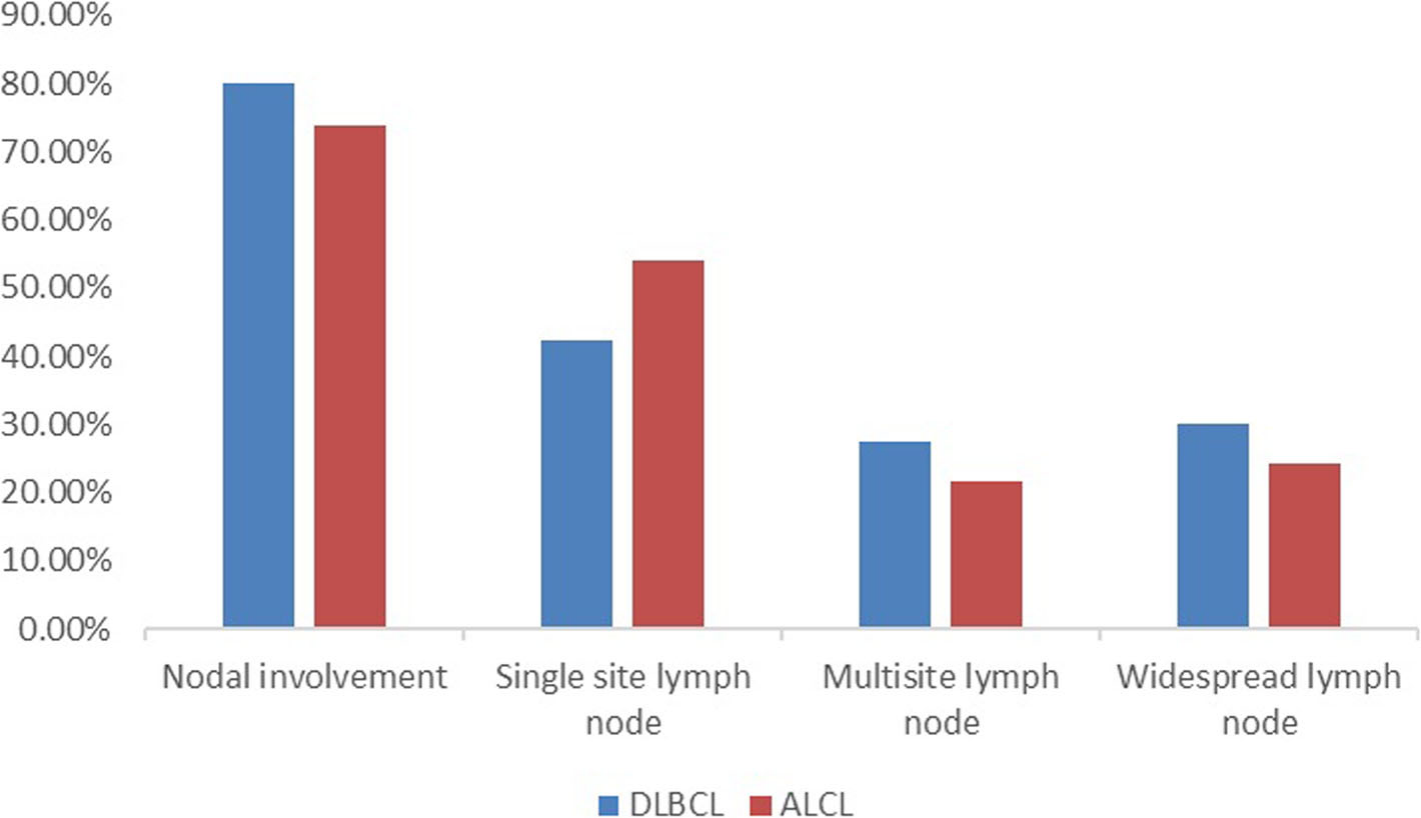
Nodal involvement and three distribution patterns in ALCL patients and DLBCL patients. No significant differences were observed in nodal involvement or the three distribution patterns between ALCL patients and DLBCL patients (*P < 0.05*)

**Fig. 2:**
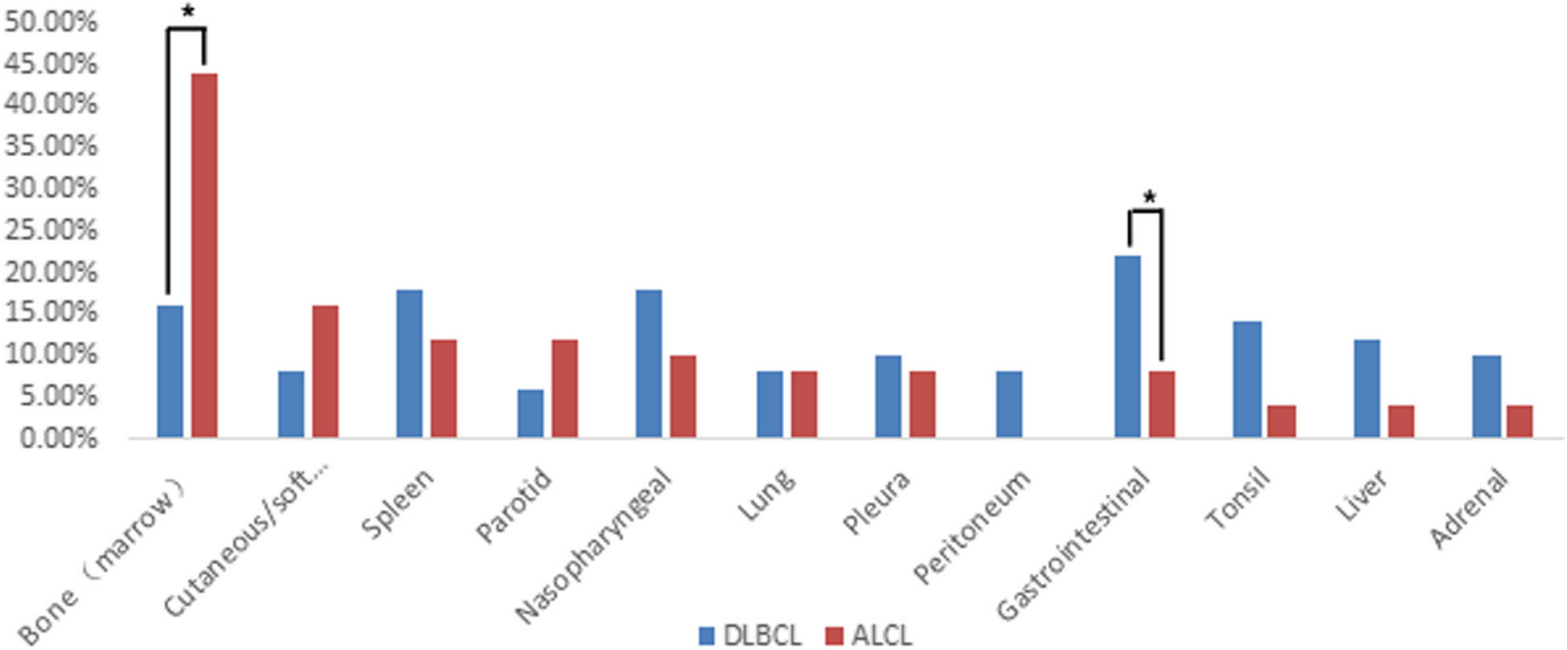
The extranodal involvement in patients with ALCLs compared to those the extranodal involvement in patients with DLBCLs. There were significant differences in tumour involvement in the bone and gastrointestinal tract between ALCL and DLBCL (*: *P <* 0.05). No significant difference in extranodal involvement in other organs was observed between ALCL and DLBCL (*P* > 0.05)

**Fig. 3:**
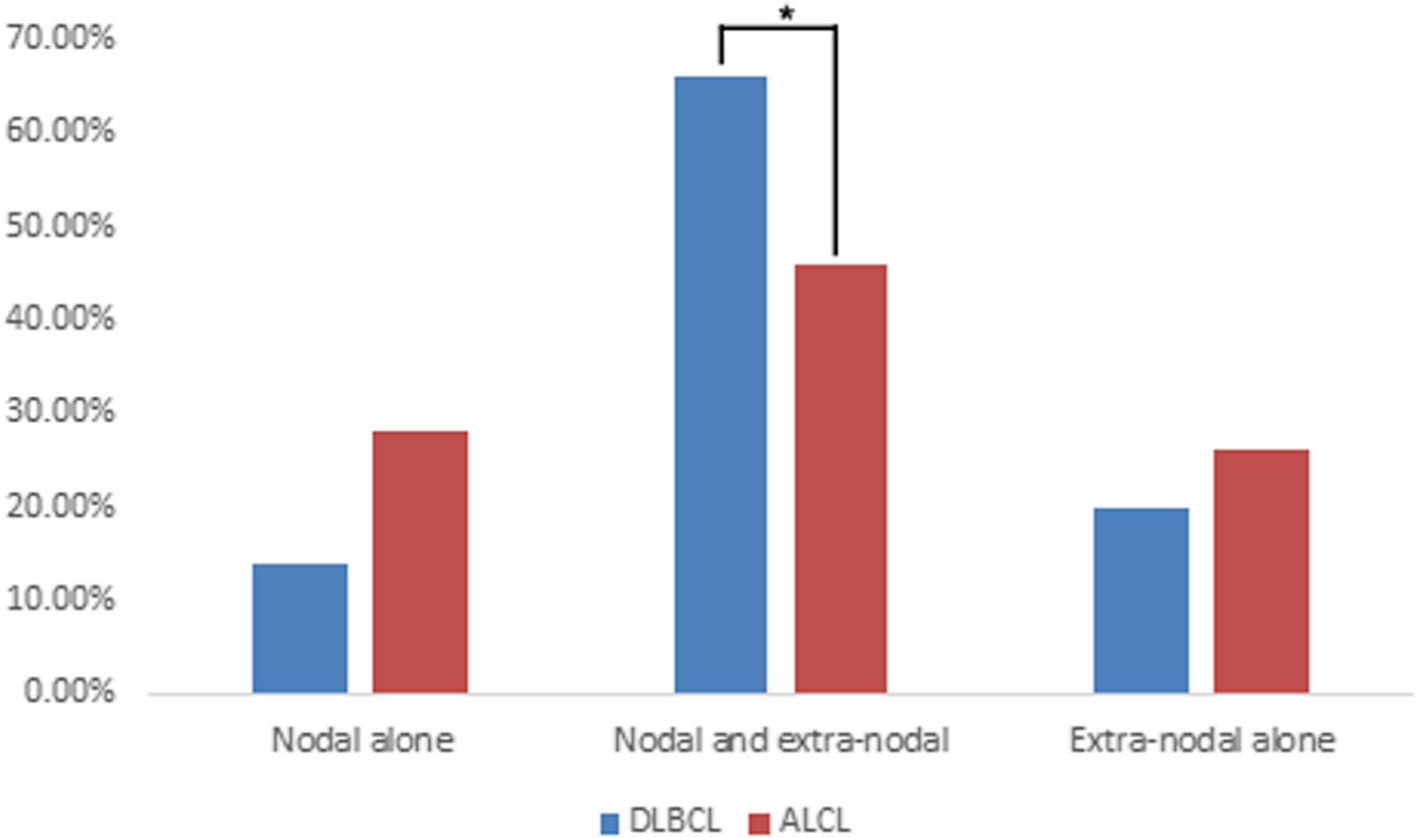
Nodal involvement alone, simultaneous nodal and extranodal involvement, and extranodal involvement alone in ALCL patients and DLBCL patients. More patients with DLBCL had simultaneous nodal and extranodal involvement compared to those with ALCL (*: *P* < 0.05)

Compared to DLBCL, ALCL lesions showed a similar positive detection rate and radioactivity uptake on ^18^F-FDG PET/CT (positive detection rate: 98.0% vs. 100%, χ^2^ = − 0.990, *P* = 1.000; SUVmax: 19.4 ± 12.6 vs. 21.9 ± 10.7, *t* = − 0.996, *P* = 0.322). Nodal involvement was found in 74.0% of ALCLs, which was similar to the 80.0% nodal involvement observed in the DLBCLs (χ^2^ = 0.508, *P* = 0.476) (Fig. 1). There was no significant difference in the mean sites of positive lymph nodes between ALCL and DLBCL (5.8 ± 5.0 vs. 5.9 ± 4.1 *t* = − 0.088, *P* = 0.930). When nodal lesions were divided into 3 patterns: single site, multisite and widespread involvement, no significant difference in nodal involvement patterns was observed between ALCL and DLBCL (χ^2^ = 0.312–1.028, *P* = 0.311–0.576) (Fig. 1). For extranodal involvement, positive lesions were found in 72.0% of ALCL and 86.0% of DLBCL (χ^2^ = 2.954, *P* = 0.086). There was no significant difference in the number of extranodal lesions between ALCL and DLBCL (2.2 ± 1.6 vs. 2.0 ± 1.2, *t* = 0.607, *P* = 0.546). However, the favoured sites of extranodal organ involvement in ALCL showed some differences from the favoured sites in DLBCL. In DLBCL patients, the gastrointestinal tract was the most commonly involved organ (11/50), followed by the nasopharynx (9/50), spleen (9/50), bone (8/50), and tonsils (7/50), while the most commonly involved organ of ALCL was bone (22/50), followed by the skin (8/50), spleen (6/50) and parotid gland (6/50) (Fig. 2). There were significant differences in gastrointestinal tract involvement and bone involvement between DLBCL and ALCL (gastrointestinal tract involvement: 11/50 vs. 4/50, respectively, χ^2^ = 3.843, *P* = 0.050; Bone involvement: 8/50 vs. 22/50, respectively, χ^2^ = − 9.333, *P* = 0.002) (Fig. 2). In the patients with ALCLs, disease that presented only as bone lesions was observed in 8 patients; on the contrary, this phenomenon was not found in any DLBCLs (8/50 vs. 0/50, χ^2^ = 8.696, *P* = 0.006). For both nodal and extranodal involvement, more DLBCL patients were found to have simultaneous nodal and extranodal involvement than ALCL patients (66.0% vs. 46.0%, χ^2^ = 4.058, *P* = 0.044) (Fig. 3).

### Differences in clinical characteristics and image manifestation of ^18^F-FDG PET/CT between ALK-positive and ALK-negative ALCL

There were no significant differences in age, sex, B symptom or Ann Arbor stage between the ALK-positive and ALK-negative groups (*P* > 0.05) (Table 1).

**Table 1:** The clinical characteristics of ALCL patients who were ALK-positive compared to those who were ALK-negative

Clinical Characteristics	Expression of ALK		*P*
	Positive	Negative	
Age (year-old)			
< 30	17	8	0.556
≥ 30	15	10	
Gender			
Male	20	15	0.123
Female	12	3	
B symptoms			
Present	22	12	0.880
Absent	10	6	
Ann Arbor stage			
I-II	7	5	0.735
III-IV	25	13	
^18^F-FDG uptake (SUVmax)	22.1 ± 14.3	15.1 ± 6.6	0.023

**Fig. 4:**
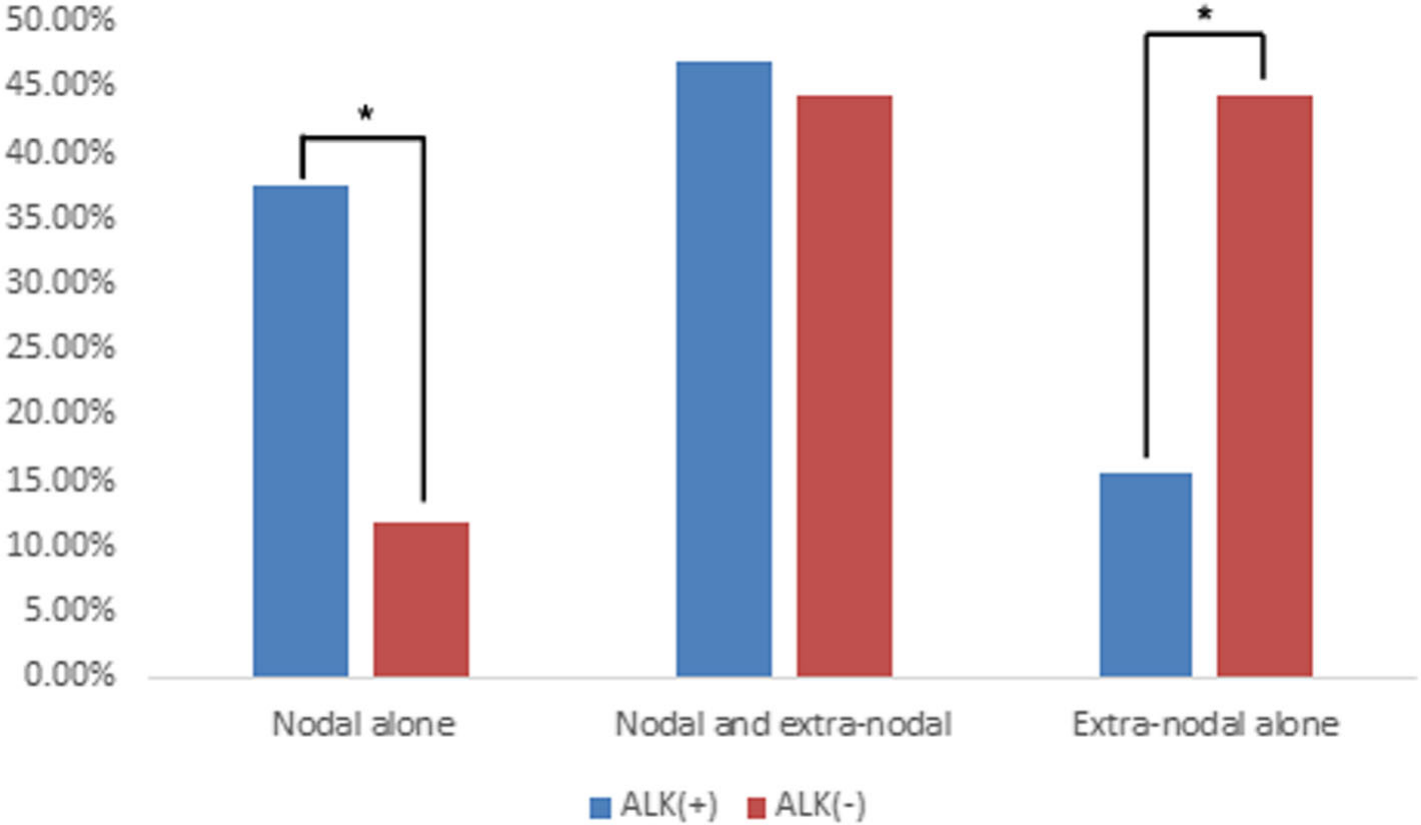
Nodal involvement alone, simultaneous nodal and extranodal involvement, and extranodal involvement alone in ALK-positive and ALK-negative ALCL patients. There were significant differences in nodal involvement alone and extranodal involvement alone between ALK-positive and ALK-negative ALCL patients (*: *P* < 0.05)

**Fig. 5:**
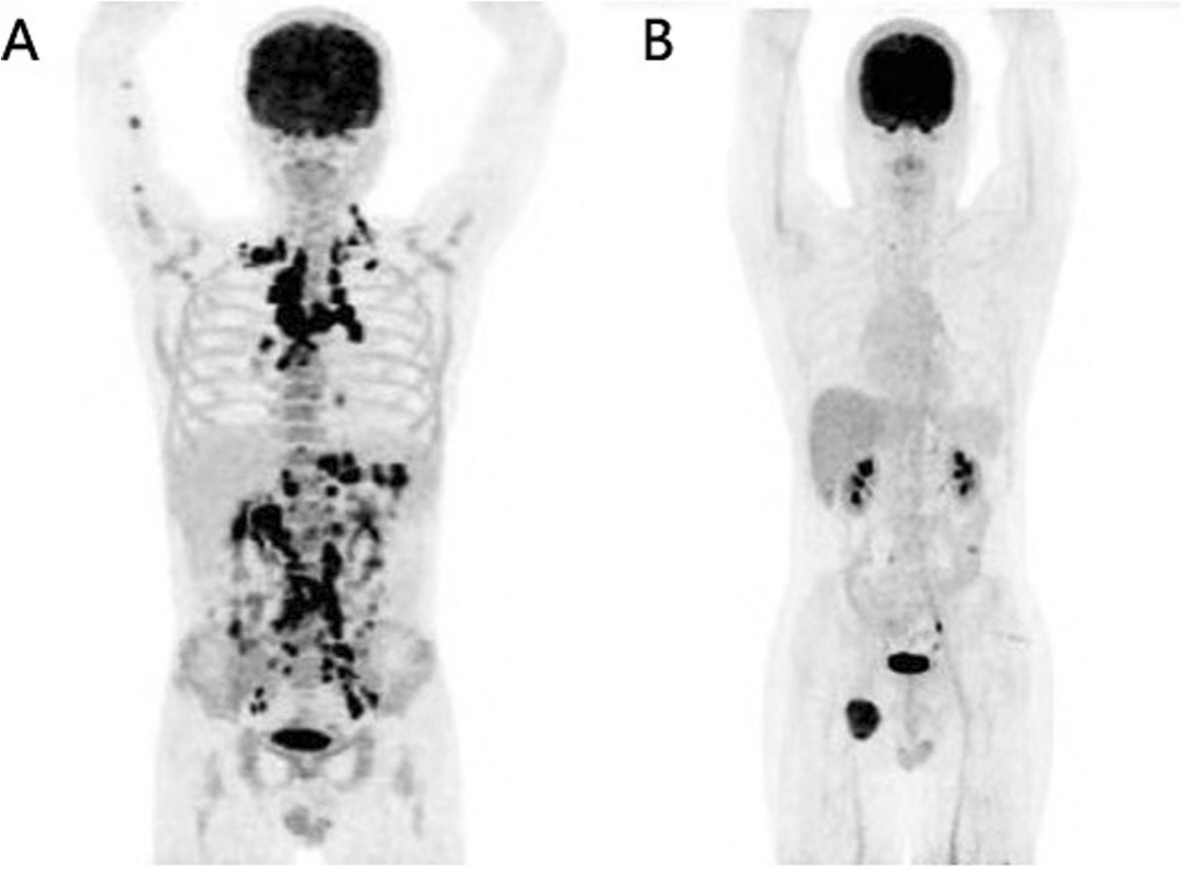
^18^F-FDG PET maximum intensity projection (MIP) images of lymph node involvement alone in two ALCL patients. Extensive involvement of systemic lymph nodes with intense ^18^F-FDG uptake (SUVmax, 16.7) was observed in a patient with ALK-positive ALCL **a**, while only a localized lesion with increased ^18^F-FDG uptake (SUVmax, 10.6) was noted in the right iliac fossa in a patient with ALK-negative ALCL (**b**)

**Fig. 6:**
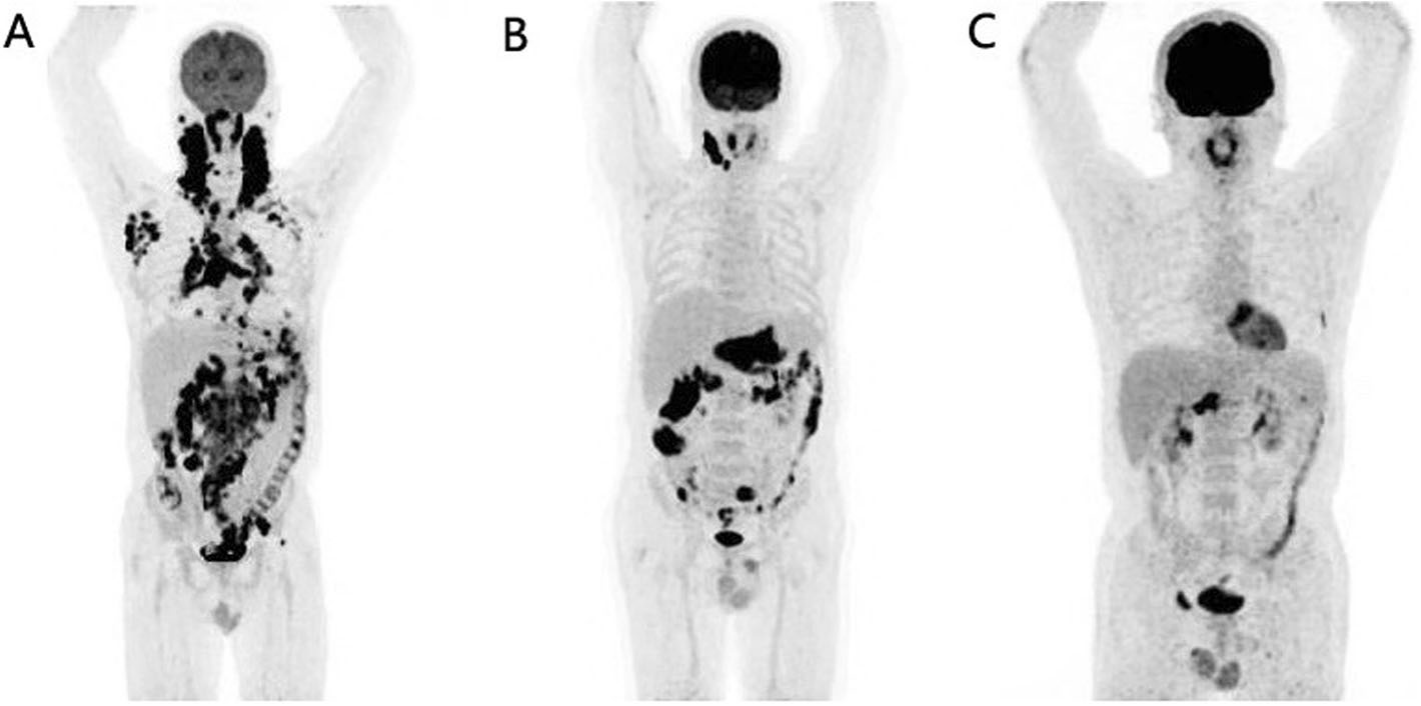
^18^F-FDG PET maximum intensity projection (MIP) images of three patients with ALK-positive ALCL (**a**) and ALK-negative ALCL (**b**, **c**). **a**: Extensive involvement of systemic lymph nodes and extranodal organs of the nasopharynx and right parotid gland were found in a patient with ALK-positive ALCL. **b**: ^18^F-FDG-avid involvement was observed in the right localized cervical lymph nodes and extranodal organs of the stomach, small intestine and colon in a patient with ALK-negative ALCL. **c**: ^18^F-FDG-avid involvement of extranodal organs alone was detected in the bones of the left 6 lateral ribs, lumbar vertebra and right superior ramus of the pubis in a patient with ALK-negative ALCL

**Fig. 7:**
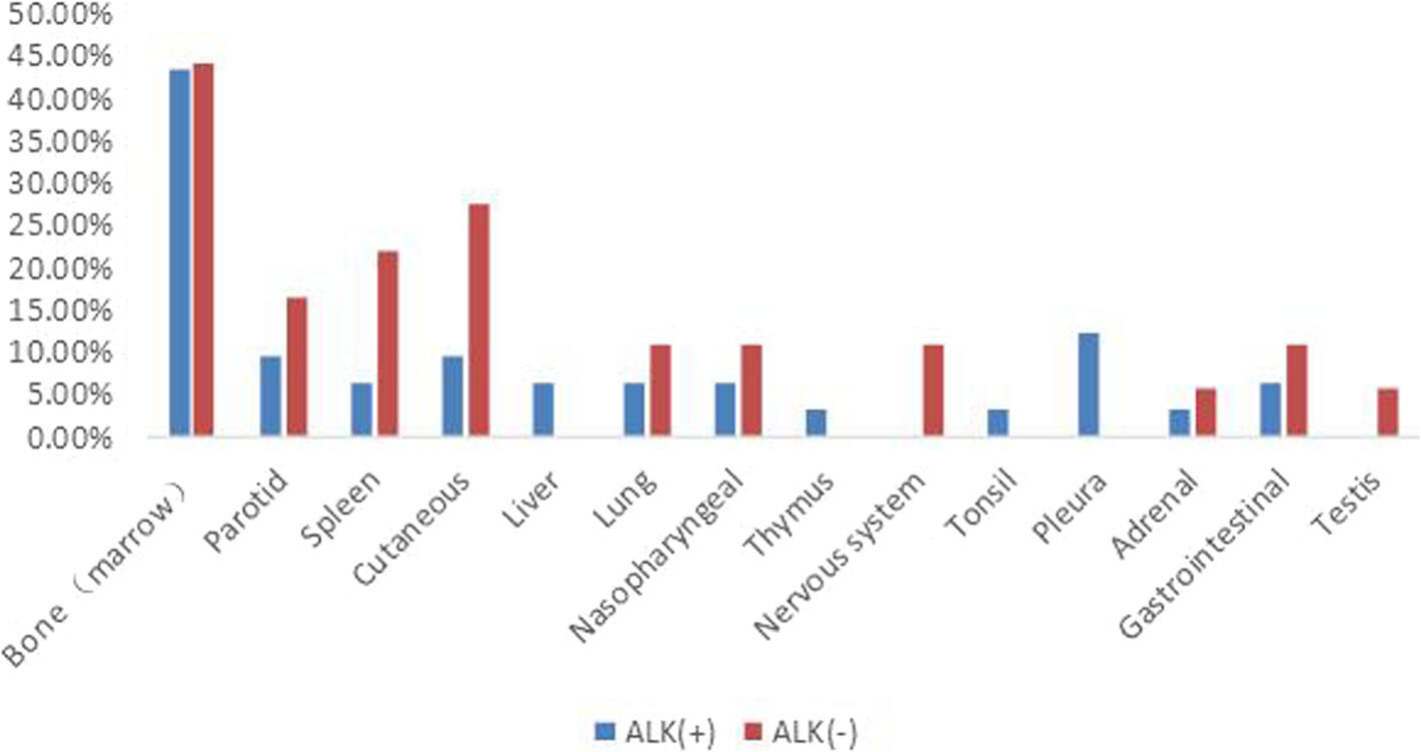
Extranodal organs involved by ALK-positive and ALK-negative ALCLs. There were no significant differences in the distribution of extranodal involvement between ALK-positive and ALK-negative ALCLs (*P* < 0.05)

All ALK-negative ALCLs were ^18^F-FDG positive, while 31/32 of ALK-positive ALCLs were ^18^F-FDG positive. More intense uptake of ^18^F-FDG was found in the ALK-positive lesions compared to that of ALK-negative lesions (SUVmax: 22.1 ± 14.3 vs. 15.1 ± 6.6, *t* = 2.354, *P* = 0.023). Regarding lesion distribution, ALK-positive ALCL was found to be more likely to involve the lymph nodes than ALK-negative ALCL; 84.3% (27/32) of ALK-positive ALCLs were observed to have positive lymph nodes compared to 55.5% (10/18) of ALK-negative ALCLs (χ^2^ = 4.973, *P* = 0.043). The number of ^18^F-FDG-avid lymph nodes was 7.0 ± 4.7 for ALK-positive ALCL, which was significantly higher than that (3.4 ± 2.1) for ALK-negative ALCL (*P* = 0.003). Nodal involvement alone was observed in 12/32 ALK-positive ALCLs, which was significantly more than the 2/18 ALK-negative ALCLs (χ^2^ = 3.979, *P* = 0.046) (Fig. 4). In ALK-negative patients with nodal involvement alone, the nodal lesions were found to be localized. However, the patients with ALK-positive (9/12) ALCL were apt to have widespread nodal lesions (Fig. 5). In those patients with nodal and extranodal involvement simultaneously, more lymph node sites were also noted in patients with ALK-positive ALCL compared to patients with ALK-negative ALCL (Fig. 6). On the other hand, ALK-negative ALCL was more likely to involve the extranodal organs than ALK-positive ALCL (88.9% vs. 62.5%, χ^2^ = 3.979, *P* = 0.046). Extranodal involvement alone was more commonly found in ALK-negative ALCL compared to ALK-positive ALCL (44.4% vs. 15.6%, χ^2^ = 4.973, *P* = 0.043) (Fig. 4). However, there were no significant differences in the organ distribution of extranodal involvement between ALK-positive and ALK-negative ALCLs (*P* < 0.05) (Fig. 7).

## Discussion

^18^F-FDG PET/CT has become a standard imaging modality in staging, treatment response evaluation and prognostic prediction for lymphoma [12–15]. It can provide some semi-quantitative indicators, such as SUVmax, that can quantify the glycolysis of tumours, and it is often reported to be associated with the tumour grade and aggressiveness [16]. ^18^F-FDG PET/CT can also show the tumour distribution in each individual and reflect the growth patterns of the tumours [17]. However, although ^18^F-FDG PET/CT has been extensively applied for diagnosing lymphoma [13], only a few papers have investigated the utility of this modality in ALCL [9, 18] and the imaging manifestations of ^18^F-FDG PET/CT for this disease have been rarely described.

The present study revealed that ALCL had intense uptake of ^18^F-FDG in tumours with a mean SUVmax of 19.5 ± 12.6. Due to the high ^18^F-FDG uptake, PET/CT had a very high sensitivity for the detection of ALCL (98.0%), which was consistent with the results reported by Lee DY, et al. [9]. High uptake of ^18^F-FDG in the lesions implies that PET/CT is a perfect modality for staging and treatment response evaluation for ALCL. In the present study, it was confirmed that ^18^F-FDG PET was more sensitive for detecting extranodal involvement compared to CT, notably for detecting bone and cutaneous involvement (*P* < 0.05). A similar result was reported by Ram-Wolff et al.; ^18^F-FDG PET was more sensitive than CT in detecting cutaneous lesions [19].

The present study demonstrated that most ALCLs (76%) presented with an advanced stage (stage III-IV) at the first visit to the doctor. ALCL commonly invaded the lymph nodes (74.0%), and the extranodal organs (72.0%). In nearly half of the patients (46.0%), simultaneous involvement of both lymph nodes and extranodal organs was found on PET/CT in the present study. Among the extranodal organs, bone was demonstrated to be the most commonly involved organ, which is inconsistent with previously published articles have shown that the skin is the most common extranodal involvement site [4, 20]. In our results, the skin was found to be the second most commonly involved extranodal site.

Although ALCL and DLBCL have different cell origins, they both have a good treatment response and prognosis, which is different from other T cell lymphoma subtypes [21, 22]. We hypothesized that ALCL and DLBCL may have some similarities in their biological behaviour that contribute to their similarities in treatment response and prognosis. The present data revealed that the biological characteristics of ALCL and DLBCL had some similarities. Both diseases presented as advanced stage in most patients at their first visit with the doctor, and both diseases had a high ^18^F-FDG uptake on PET/CT (SUVmax: 19.4 ± 12.6 vs. 21.9 ± 10.7, *t* = − 0.996, *P* = 0.322) with similar distributions of nodal involvement. Therefore, ^18^F-FDG PET/CT may play a similar important role in ALCL and DLBCL. Despite the above similarities, some differences were observed between ALCL and DLBCL. In the present study, it was found that ALCL was more likely to involve the bone. In contrast, the most commonly involved extranodal organ in DLBCL was the gastrointestinal tract, which was similar to the data reported by Prasanna Ghimire [23]. In addition, more DLBCL patients were found to have simultaneous involvement of nodal and extranodal sites than ALCL patients (66.0% vs. 46.0%, χ^2^ = 4.058, *P* = 0.044). To the best of our knowledge, there have been no prior studies comparing these similarities and differences between ALCL and DLBCL. Therefore, the findings in the present study may be useful to improve the understanding of the biological characteristics of this rare subtype of lymphoma.

Translocations involving the anaplastic lymphoma kinase (ALK) and nucleophosmin (NPM) were first identified in ALCLs [24, 25]. Subsequently, ALK translocations were also found in other cancers, such as lung cancers [26]. The present study found that ALK-positive ALCL had a higher ^18^F-FDG uptake than that of ALK-negative ALCL (SUVmax: 22.1 ± 14.3 vs. 15.1 ± 6.6, *t* = 2.354, *P* = 0.023), which was consistent with the results reported by Lee DY [9]. Similarly, ALK-positive lung cancers were also observed to have a higher SUVmax than ALK-negative [27, 28] lung cancers. Untreated extranodal natural killer/T-cell lymphomas of the head and neck with a higher glycolysis are often reported to be resistant to treatment and have a poor prognosis [29]. However, the present study implied that ALCL is different from natural killer/T-cell lymphomas of the head and neck. The intensity of ^18^F-FDG uptake in ALCL tumours may not be an indicator of treatment response and prognosis because ALK-positive ALCL was always reported to have a better prognosis than ALK-negative ALCL [1, 5–7, 30]. Experimental results reported by McDonnell SR may explain why ALK-positive ALCL had a higher ^18^F-FDG uptake [31]. Their study demonstrated that NPM-ALK induced a metabolic shift towards aerobic glycolysis. The metabolic shift was mediated through ALK phosphorylation of the tumour-specific isoform of pyruvate kinase (PKM2), resulting in decreased enzymatic activity. As a result, oxidative phosphorylation decreased, and aerobic glycolysis increased, coincident with increased lactate production and biomass production. The study by Ma Y also revealed that the echinoderm microtubule-associated protein-like 4-anaplastic lymphoma kinase (EML4-ALK) was coupled to overexpression of hexokinase II (HK2), one of the rate-limiting enzymes of the glycolytic pathway, which induced hyperactive glycolysis in EML4-ALK-positive lung cancer [32].

In the present study, in addition to differences in ^18^F-FDG uptake, we also found that there were some differences in nodal and extranodal involvement between ALK-positive and ALK-negative patients. ALK-positive ALCL was observed to be more likely to involve the lymph nodes than ALK-negative ALCL (84.3% vs. 55.5%, χ2 = 4.973, *P* = 0.043) and had more sites of positive lymph nodes (7.0 ± 4.7 vs. 3.4 ± 2.1, *t* = 3.247, *P* = 0.003). In contrast, ALK-negative ALCL was found to be more prone to involve the extranodal organs compared to ALK-positive ALCL (88.9% vs. 62.5%, χ2 = 3.979, *P* = 0.046). Therefore, we suggested that the less favourable survival outcomes of ALK-negative ALCL [3, 7] may be due to the more extensive extranodal involvement, which was also proposed by Nguyen et al. [4].

There are some limitations to the present study. First, the patient sample size was small, and further research is warranted to confirm our findings. Second, due to the shortcomings of retrospective studies, a prospective study may be more useful to discover the imaging characteristics of ALCL.

## Conclusions

The present study demonstrated that ALCL is a systemic, ^18^F-FDG-avid tumour. ALCL and DLBCL had some similarities, such as advanced stage in most patients, high ^18^F-FDG uptake and a similar distribution of nodal lesions. However, ALCL and DLBCL had some differences in extranodal involvement, especially in the bone and the gastrointestinal tract. The present study also found that ALCL with ALK-positive cells had a higher ^18^F-FDG uptake than ALK-negative ALCL. In addition, ALK-positive ALCL was more likely to involve the lymph nodes and had more sites of lymph node positivity. In contrast, ALK-negative ALCL was more prone to involve the extranodal organs. The findings in the present study may be useful to improve the understanding of the biological characteristics of this rare subtype of lymphoma.

## Data Availability

Not applicable.

## Abbreviations

ALCL: Anaplastic large cell lymphoma
ALK: Anaplastic lymphoma kinase
CT: Computed tomography
DLBCL: Diffuse large B cell lymphoma
EML4-ALK: The echinoderm microtubule-associated protein-like 4-anaplastic lymphoma kinase
NHL: Non-Hodgkin’s lymphoma
NPM: Nucleophosmin
OSEM: Ordered subset maximum expectation iterative method
PET: Positron emission tomography
ROI: Region of interest
SUV_max_: The maximal standardized uptake value
WHO: World Health Organization
